# A Case of Purulent Sub-Arachnitis, Probably Metastatic

**Published:** 1883-07

**Authors:** George Thompson

**Affiliations:** Medical Superintendent of the Bristol Lunatic Asylum, Stapleton


### A CASE OF PURULENT SUB-ARACHNITIS, PROBABLY METASTATIC. By GEORGE THOMPSON, M.D., L.R.C.P., Medical Superintendent of the Bristol Lunatic Asylum, Stapleton.

Jane W., æt. 56, single, a manager of a registry office at Clifton, was admitted to the Wilts County Asylum, at Devizes, on the 8th of September, 1880.

Without giving any warning she disappeared from her home at Clifton, and was picked up by the authorities of an Union in Wiltshire, and, as she could give no account of herself, sent to the County Asylum. While there she at first suffered from mania. She was unable to give an account of herself, or any reliable information as to her past life. She said she had been tempted by the devil to destroy herself, and believed she was going to be severely tortured. Fourteen days later she had settled down, and complained of pain in her head. Bromide of potassium was prescribed for her.*

She was admitted to the Bristol Asylum on the 3rd of June, 1881. She was in fair bodily health, but was evidently suicidally disposed.

From time to time she complained of pain in the head, referring it to the occiput. No especial note was made of her until April 2nd, 1883, when she complained of sickness and diarrhœa.

* The above is abridged from a copy of the notes in the case-book of the Wilts Asylum, kindly supplied to me by Mr. Bowes, the Medical Superintendent.

On the evening of the 2nd April her temperature was found to be 102.8, and on the following morning 103.4, pulse 120, respiration 30. Her right lung was dull at the back, and some coarse crepitation was heard, with increase of vocal fremitus.

She passed through an attack of "single" pneumonia and appeared to be recovering, when, on the 9th instant, the temperature suddenly rose. Albumen appeared in the urine in large quantities, though no casts were found under the microscope. She became gradually insensible, and at mid-day, on the following day, she died, her temperature having in the meantime reached 106°.

But for the examination of the organs after death her dying would have been attributed to renal congestion.

At the *post mortem* examination the left lung and the liver were found to be quite healthy. The right lung was in a state of grey hepatization, and the kidneys and spleen were intensely congested. The brain was only slightly congested, but the whole of the arachnoid space was filled out with a semi-solid matter, which was found to be lowly organised lymph and pus. This was most marked on the convolutions at the upper aspect of the brain, gradually thinning off until the under surface of the brain was reached. At the pons and medulla some free pus was seen. The pia mater infiltrated with this matter was easily stripped from the surface of the brain, and without laceration of the brain substance. If there was any difference of intensity, there was more of the purulent lymph on the left side.

Opacities of the arachnoid and sub-arachnoid effusions are by no means rare in the cases which come on the *post mortem* examination table of a large asylum, where examinations are the rule and not the exception; but with an experience extending over nearly a thousand examinations, I have never before met with such a condition as that I have briefly stated above.

The older writers—Prichard, Spurzheim, Noble and others—do not seem to have recognised such a condition, and even more modern writers, such as Sankey, whose work is based entirely on personal observations, Feuchtersleben and Griesinger do not mention such a condition. The most modern writers, in which I include Ross, Althaus and Long Fox, do refer to it. Ross even describes it as pneumonic metastasis, and this case may, I think, fairly be ranked under that class. It is more from its extreme rarity than from its especial interest that I have brought the case before you to-night. I was tempted to give it a name, which would make its position and nature more precise: hence the title of this paper.

*(Read before the Bristol Medico-Chirurgical Society, May 9th, 1883.)*

